# High initial FSH dosage reduces the number of available cleavage-stage embryos in a GnRH-antagonist protocol: Real-world data of 8,772 IVF cycles from China

**DOI:** 10.3389/fendo.2022.986438

**Published:** 2022-10-17

**Authors:** Xiu Luo, Li Pei, Yao He, Fujie Li, Wei Han, Shun Xiong, Shubiao Han, Jingyu Li, Xiaodong Zhang, Guoning Huang, Hong Ye

**Affiliations:** ^1^ Center for Reproductive Medicine, Chongqing Health Center for Women and Children, Chongqing, China; ^2^ Key Laboratory of Human Embryo Engineering, Chongqing Maternal and Child Health Care Hospital, Chongqing, China

**Keywords:** *In vitro* fertilization, GnRH antagonist, FSH dose, cleavage-stage embryos, real world evidence

## Abstract

To evaluate the relationship between the initial follicle stimulating hormone (FSH) dose and the number of available cleavage-stage embryos in *in vitro* fertilization (IVF) cycles.We included 8772 fresh IVF cycles using a GnRH antagonist protocol at the Genetic and Reproductive Institution of Chongqing, P. R. China, from January 2016 to June 2021.Univariate linear regression was used to evaluate the associations between the initial FSH dosage (≤ 150, 187.5–200, 225, 250, or 300 IU) with the number of available cleavage-stage embryos on day 3. A two-factor linear regression model was applied to calculate the threshold effect of the initial FSH dosage on the number of available cleavage-stage embryos based on a smoothing plot. The initial FSH dose was negatively correlated with the number of available cleavage-stage embryos, independent of female age, body mass index, infertility factors, duration of infertility, anti-Müllerian hormone and basal FSH levels, antral follicle count and the proportions of patients with poor ovarian response or polycystic ovarian syndrome. Using a two-factor linear regression model, we calculated the inflection point to be 200 IU of FSH. The relationship between the initial FSH dose and the number of available cleavage-stage embryos was nonlinear. The initial FSH dose was negatively associated with the number of available cleavage-stage embryos when the initial FSH dose was > 200 IU. Therefore, clinicians should try to avoid unnecessarily increasing the initial FSH dose.

## Introduction

Controlled ovarian stimulation (COS) is the core of assisted reproductive technology (ART). Previous studies have shown that the more embryos available for transfer, the higher are the cumulative pregnancy and live birth rates (1). To attain higher cumulative live birth rates, ART clinics usually use gonadotropins in supra-physiologic doses for COS to produce more mature oocytes and enough embryos for transfer. During a cycle of *in vitro* fertilization/intracytoplasmic sperm injection (IVF/ICSI), women receive daily doses of follicle-stimulating hormone (FSH) to induce multi-follicular development in the ovaries. Generally, the dose of FSH is associated with the quantity and quality of oocytes retrieved. However, the relationships between the initial FSH dose and the quantity and quality of oocyte remains controversial. A meta-analysis including 22 randomized controlled trials (n = 6088) did not find that tailoring the FSH dose to any particular ovarian reserve population (low, normal, or high), influenced the numbers of oocytes retrieved or the live birth/ongoing pregnancy rates (2). In addition, two retrospective analyses of large samples showed a negative correlation between total FSH dosage and the number of oocytes retrieved (3) or live birth rate (4, 5), suggesting that an increase in total FSH affects the quantity and quality of oocytes and influences live birth outcomes. Therefore, ART clinics are increasingly interested in whether high doses of FSH affect the quantity and quality of oocytes, and exactly how high the dose affects. As early as the 1980s, dose-response studies in cattle showed that there is a maximum response plateau for the FSH dose of ovulation stimulation and that FSH doses above this plateau result in a decrease in follicle number, estradiol (E2) levels, the number of oocytes received, number of fertilized ova, the number of available embryos (6–15)] and an increase in the number of degenerated embryos (10). In human ART clinics, similar studies have shown that high doses of FSH do not improve the number of mature oocytes retrieved or the outcome of assisted conception (16–18). Then how to determine the initial FSH dose during ovarian stimulation is very important to guide the clinical process. Is it possible to find a maximal response plateau for initial FSH dose in humans, thus improving assisted conception outcomes?

The objective of this study was to examine the relationship between the initial FSH dose and the number of available cleavage-stage embryos in ART cycles with use of a large database.

## Materials and methods

### Study participants

This was a retrospective cohort study. The study population included fresh IVF/ICSI cycles using at least one autologous oocyte at the Genetic and Reproductive Institution of Chongqing, P. R. China, from January 2016 to June 2021. All patients underwent a GnRH antagonist protocol for COS. All procedures of this study were approved by the Institutional Review Board of Chongqing Health Center for Women and Children(2021-RGI-12). The requirement for patient informed consent was waived by the Institutional Review Board because the retrospective cohort study involved existing data and records at the time of investigation, and did not retain personal identifiers in the collected information. The initial FSH treatment included recombinant FSH and urinary (u)FSH, and did not include any luteinizing hormone (LH) activity. Polycystic ovarian syndrome (PCOS) was defined according to the Rotterdam ESHRE/ASRM-Sponsored PCOS consensus workshop group (19). Poor ovarian reserve (POR) was defined as anti-Müllerian hormone (AMH) <1.2 mIU/mL or antral follicle count (AFC) <5 (groups 3 and 4 in the POSEIDON criteria) (20).

### Ovarian stimulation and ART procedures

On days 2 or 3 of menstruation, the women received 100–300 IU per day recombinant FSH (rFSH, Puregon, MSD, America; Gonal-f, Merk, Germany; Jinsaiheng, GenSci, P. R. China) or Urinary FSH (uFSH, Lishengbao, Livzon (Group) Pharmaceutical Factory, P. R. China) up to the day of human chorionic gonadotropin (hCG) ovulation trigger, depending on age, BMI, basal FSH and AMH and AFC levels. The FSH dose was adjusted according to hormonal assessment and ultrasound monitoring after 4 or 5 days. Women received 0.25 mg GnRH antagonist daily (Centrotide, Merck, Germany; Orgalutran, MSD, America) from simulation day 5 or on the day that the dominant follicle diameter reached 12–14 mm or E2 levels > 600 pg/ml or LH levels > 10 IU/L up to the day of hCG administration. When at least three follicles measured ≥ 17 mm or two follicles measured ≥ 18 mm in diameter, patients received their last GnRH antagonist injection in the morning and final follicular maturation was induced the same evening by 250 μg recombinanthCG (Ovidrel, Serono, Germany). If there were more than 19 follicles ≥ 11mm in diameter on the day of rhCG administration, final follicular maturation was induced the same evening by 0.2 mg GnRH agonist (Diphereline, Ipsen/Decapeptyl, Ferring, Germany) to avoid the occurrence of ovarian hyper-stimulation syndrome (OHSS).

Oocyte retrieval took place 36–38 h after the rhCG trigger by transvaginal ultrasound-guided single lumen needle aspiration. ICSI was performed only in cases with severe male factor or previous fertilization failure. Embryo quality was evaluated for all available embryos on day 3 of culture by an experienced embryologist. Embryos graded as grade 1 (6–10 cells, no fragmentation and equal blastomere size), grade 2 (with slightly uneven blastomeres and/or cytoplasmic fragments up to 20%), grade 3 (with uneven blastomeres and/or cytoplasmic fragments of 20–30%) qualified as available cleavage-stage embryos on day 3 after oocyte retrieval (21).

### Statistical analysis

Continuous variables are expressed as the mean ± standard deviation, and categorical variables were expressed in frequency or as a percentage. One-way analysis of variance (ANOVA for normally distributed data), Kruskal–Wallis test (for skewed distributions) and chi-squared tests (for categorical variables) were used to determine any statistical differences between the means and proportions of the groups. Univariate linear regression model was used to evaluate the associations between the initial FSH dosage (≤ 150, 187.5–200, 225, 250, or 300 IU) with the number of available cleavage-stage embryos on day 3. Both unadjusted and multivariable adjusted models were used. We also used a generalized additive model (GAM) to identify any nonlinear relationship. If a nonlinear correlation was observed, a two-factor linear regression model was applied to calculate the threshold effect of the initial FSH dosage on the number of available cleavage-stage embryos in terms of the smoothing plot. When the initial FSH dosage on the number of available cleavage-stage embryos on day 3 appeared obvious in the smoothed curve, a recursive method was used to calculate the inflection point, where the maximum model likelihood was used. All of the data were analyzed with the use of the statistical packages R (The R Foundation; http://www.R-project.org; version 3.6.3) and EmpowerStats (http://www.empowerstats.net, X&Y solutions, Inc. Boston, MA, USA).

## Results

**Figure 1:**
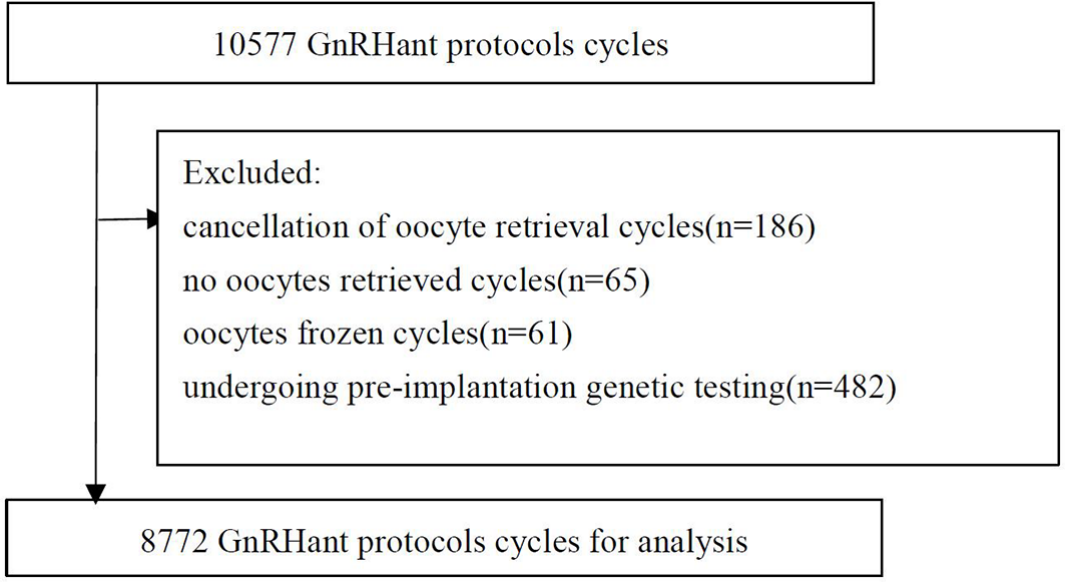
Flow chart.

There were 10,577 GnRH antagonist protocol cycles. The following cycles were excluded: 186 with cancellation of oocyte retrieval; 65 with no oocytes retrieved; 61 frozen oocyte cycles; 482 cycles for couples undergoing pre-implantation genetic testing, and 1011 from patients with chromosomal abnormalities. Ultimately, 8772 cycles were included in the analysis (**Figure 1**).

### Baseline characteristics of participants

The mean age of the female patients was 33.1 ± 5.1 y; mean BMI was 22.3 ± 3.0 kg/m^2^; mean weight was 55.7 ± 8.1 kg; mean AMH was 3.6 ± 4.2 mIU/mL; mean AFC was 7.8 ± 4.9, mean number of oocytes retrieved was 9.5 ± 6.9, mean number of cleavage-stage embryos on day3 was 4.0 ± 3.7. There were 1046 patients with PCOS (11.92%) and 3827 with POR (43.63%). We divided the cycles into five groups according to the starting dose of FSH: ≤ 150, 187.5–200, 225, 250, or 300 IU. Baseline characteristics are listed in **Table 1**. Age, BMI, duration of infertility, the proportion of patients with POR, and the total FSH dosage increased gradually with increasing initial FSH doses, and the proportion of patients with PCOS, AMH, AFC, the numbers of oocytes retrieved and available cleavage-stage embryos decreased gradually (all *P* <.001).

**Table 1:** Baseline Characteristics of participants (n=8772).

Characteristics	initial FSH dosage (IU)					
	≤150 (n=2259)	187.5-200 (n=450)	225 (n=1138)	250 (n=456)	300 (n=4469)
AGE (years)	28.6 ± 3.1	30.8 ± 3.56	32.4 ± 4.1	33.3 ± 4.0	35.9 ± 4.5
BMI (kg/m2)	21.6 ± 2.9	22.60 ± 3.55	22.05 ± 2.92	22.45 ± 3.11	22.75 ± 2.99
Duration of infertility(years)	4.4 ± 2.8	4.8 ± 3.1	5.5 ± 3.8	5.6 ± 4.1	6.4 ± 4.8
bFSH (miu/ml)	5.0 ± 1.3	5.1 ± 1.5	5.8 ± 1.7	5.9 ± 1.7	6.6 ± 2.5
AMH (ng/ml)	8.7 ± 4.8	4.9 ± 3.4	2.90 ± 2.3	2.0 ± 1.4	1.2 ± 1.0
AFC (n)	13.6 ± 4.8	10.9 ± 3.9	8.1 ± 2.9	6.6 ± 2.3	4.6 ± 1.9
initial FSH dosage (IU)	143.6 ± 11.3	195.4 ± 6.1	225.0 ± 0.0	250.0 ± 0.0	300.0± 0.0
Total FSH dosage (IU)	1374.1 ± 422.2	1709.3 ± 342.3	2011.4 ± 392.5	2198.5 ± 406.0	2730.2 ± 501.0
Infertility factors	Proportion of patients ,n (%)				
Tubal disease	1568(69.41)	310(68.89)	850(74.69)	341(74.78)	3459(77.40)
Ovulatory disorder	233(10.31)	29(6.44)	40(3.51)	12(2.63)	58(1.30)
Endometrosis	95(4.21)	27(6.00)	88(7.73)	45(9.87)	434(9.71)
Unexplained	74(3.28)	15(3.33)	39(3.43)	12(2.63)	130(2.91)
Male factor	208(9.21)	43(9.56)	73(6.41)	30(6.58)	241(5.39)
Other	81(3.59)	26(5.78)	48(4.22)	16(3.51)	147(3.29)
PCOS(%)	38.29	18.44	5.36	1.97	0.60
POR(%)	1.20	7.78	22.58	38.38	74.51
NO. of oocytes retrieved(n)	15.5 ± 7.9	14.2 ± 7.1	10.0 ± 5.3	8.7 ± 4.7	5.8 ± 3.9
NO. of available cleavage embryos on day3(n)	6.6 ± 4.7	5.7 ± 4.5	4.1 ± 3.0	3.5 ± 2.9	2.4 ± 2.0

Data presented as mean ± SD or n (%), unless otherwise stated. BMI, body mass Index; AMH, anti-müllerian hormone; bFSH, basal follicle-stimulating Hormone; AFCm, antral follicle counts; PCOS, polycystic ovary syndrome; POR, poor ovarian reserve.

### Univariate analysis

The results are shown in **Table 2**. These showed that initial FSH dosage, age, BMI, duration of infertility, bFSH level, weight, endometriosis and POR were negatively associated with the number of available cleavage-stage embryos; AMH, AFC, PCOS and ovulatory disorders were associated with more cleavage-stage embryos available.

**Table 2:** Univariate analysis for the number of available cleavage embryos on day 3.

	Statistics	Effect size (β)	*P*
initial FSH dosage(per 75 IU)	3.2 ± 0.9	-2.03 (-2.11, -1.96)	<0.0001
Age (years)	33.1 ± 5.1	-0.20 (-0.21, -0.18)	<0.0001
BMI (kg/m^2^)	22.3 ± 3.0	-0.03 (-0.06, -0.01)	0.0069
Duration of infertility (years)	5.6 ± 4.2	-0.10 (-0.12, -0.08)	<0.0001
bFSH (miu/ml)	6.0 ± 2.2	-0.48 (-0.51, -0.44)	<0.0001
AMH (ng/ml)	3.6 ± 4.2	0.43 (0.42, 0.45)	<0.0001
AFC (n)	7.8 ± 4.9	0.38 (0.36, 0.39)	<0.0001
Weight (kg)	55.7 ± 8.1	-0.01 (-0.02, -0.00)	0.0439
Ovarian reserve , n (%)			
Normal	3899(44.45)	ref	
PCOS	1046(11.92)	2.41 (2.19, 2.64)	<0.0001
Poor ovarian reserve	3827(43.63)	-2.48 (-2.62, -2.33)	<0.0001
Infertility factors , n (%)			
Tubal disease	6511 (74.42)	ref	
Ovulatory disorder	371 (4.24)	2.14 (1.77, 2.52)	<0.0001
Endometrosis	686 (7.84)	-0.93 (-1.22, -0.65)	<0.0001
Unexplained	271 (3.10)	-0.24 (-0.68, 0.19)	0.2755
Male factors	593 (6.78)	-0.09 (-0.39, 0.22)	0.5742
Other	317 (3.62)	-0.16 (-0.56, 0.25)	0.4519

Ref, reference.

### Relationship between initial FSH dose and the number of available cleavage-stage embryos

We used univariate linear regression models to assess the relationship between the initial FSH dose and the number of available cleavage-stage embryos. We show the unadjusted and adjusted model in **Table 3**. In the crude model, the initial FSH dose was negatively associated with the number of available cleavage-stage embryos (β = –2.03; 95% confidence interval, CI, –2.11 to –1.96, *P* <.0001). In the adjusted model, the results did not change significantly (β = –0.35; 95% CI, –0.5 to –0.2, *P* <.001). For sensitivity analysis, we also analyzed the initial FSH dose in five groups and found the same significant trend (*P* <.001 for trend).

**Table 3:** Relationship between initial FSH dosage (per 75 IU) and the number of available embryos on day3 in different models.

outcome	Crude model		adjusted model	
	β(95%CI)	*P-value*	β(95%CI)	*P*
initial FSH dosage (per 75 IU)	-2.03 (-2.11, -1.96)	<0.0001	-0.35 (-0.50, -0.20)	<0.0001
initial FSH dosage group				
≤150 IU	*Ref*		*Ref*	
187.5~200 IU	-0.96 (-1.28, -0.64)	<0.0001	0.39 (0.06, 0.72)	0.0201
225 IU	-2.56 (-2.79, -2.34)	<0.0001	-0.24 (-0.53, 0.04)	0.0878
250 IU	-3.12 (-3.44, -2.80)	<0.0001	-0.34 (-0.72, 0.03)	0.0727
300 IU	-4.21 (-4.37, -4.05)	<0.0001	-0.67 (-0.99, -0.35)	<0.0001
*P* for trend	<0.001		<0.001	

Adjusted for female age, BMI, weight, infertility factors, duration of infertility, AMH, bFSH, AFC, the proportion of POR and PCOS. CI, confidence interval.

### Analyses of nonlinear relationships

**Figure 2:**
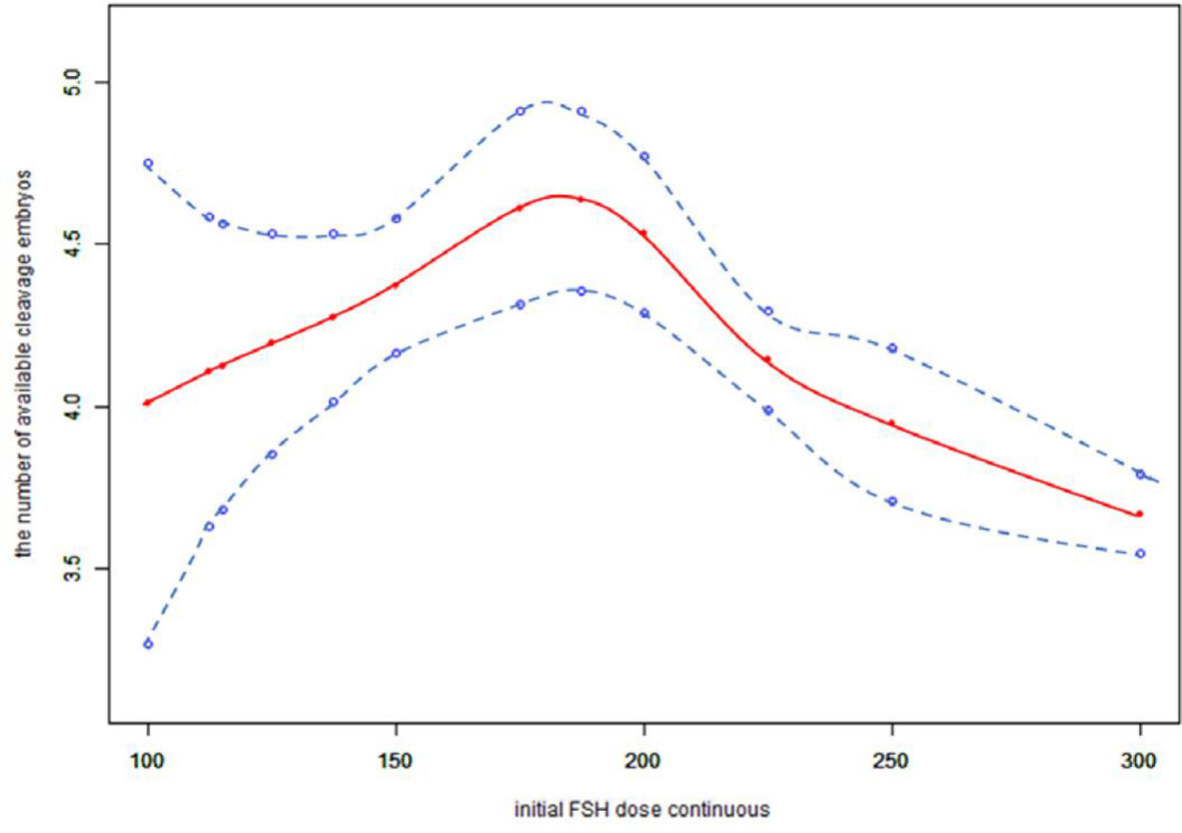
Association between the initial FSH dose and the number available cleavage embryos on day3. A threshold, nonlinear association between the initial FSH dose and the number available cleavage embryos was found (p<0.0001) in a generalized additive model (GAM). Solid red line represents the smooth cure fit between variables. Blue bands represent the 95% of confidence interval from the fit. All adjusted for female age, BMI, weight, infertility factors, duration of infertility, AMH, bFSH, AFC, the proportion of POR and PCOS.

Because the initial FSH dose was a continuous variable, it was necessary to analyze the nonlinear relationships. We found that the relationship between the initial FSH dose and the number of available cleavage-stage embryos was non-linear (**Figure 2**). Using a two-factor linear regression model, we calculated that the inflection point was 200 IU of FSH. Above the inflection point, the number of available cleavage-stage embryos decreased by 0.7 per 75 IU increase in the initial FSH dose (effect size β = 0.70; 95% CI –0.88 to –0.51 and *P* <.0001). However, we observed no relationship between the initial FSH dose and the number of available cleavage-stage embryos to the left of the inflection point (effect size β = –0.22; 95% CI –0.12 to 0.56, *P* = .21; **Table 4**).

**Table 4:** Threshold Effect Analysis of initial FSH dose (per 75 IU) and the number of available cleavage embryos using Piece-wise Linear Regression.

initial FSH dose	Effect size (β)	95%CI	*P*
≤200 IU	0.22	-0.12, 0.56	0.21
>200 IU	-0.70	-0.88, -0.51	<0.0001

Adjusted for female age, BMI, weight, infertility factors, duration of infertility, AMH, bFSH, AFC, the proportion of POR and PCOS.

Effect: the number of available cleavage embryos on day 3 Cause: initial FSH dose(per 75 IU).

**Figure 3:**
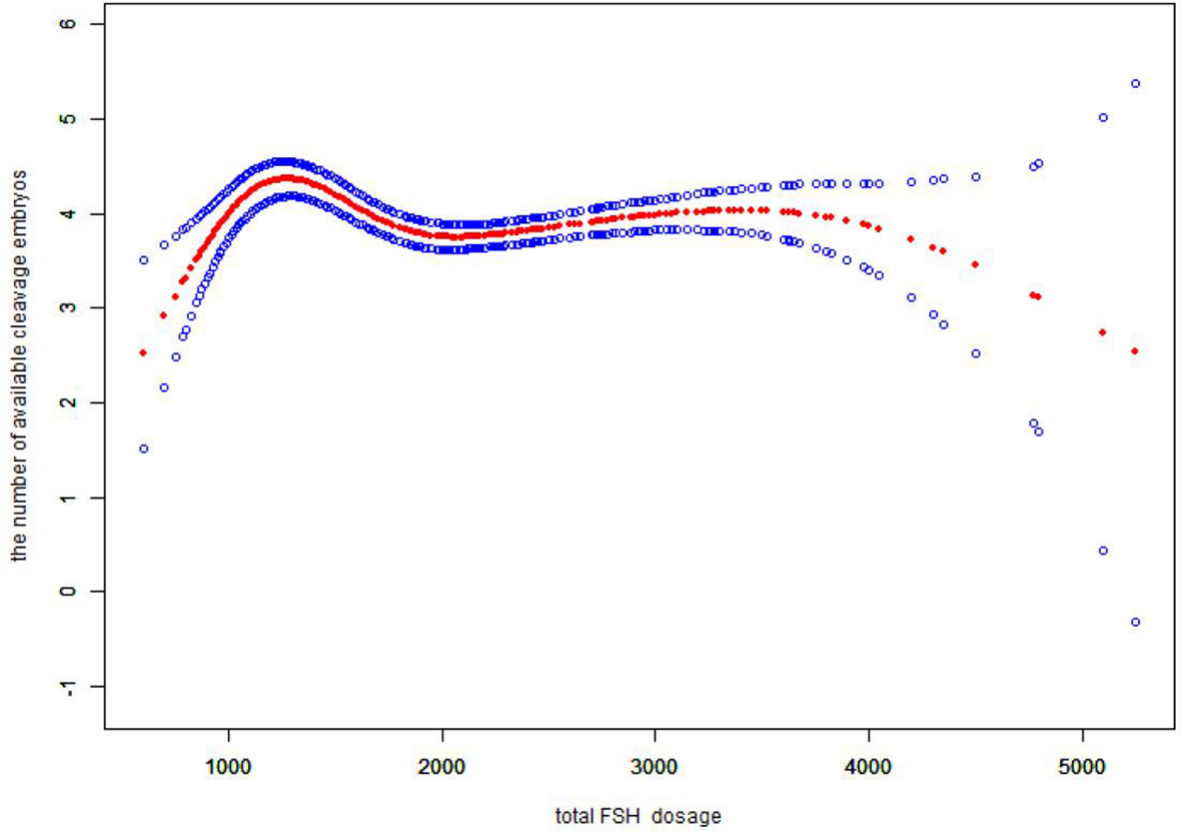
Association between the total FSH dosage and the number available cleavage embryos on day3. A threshold, nonlinear association between the total FSH dosage and the number available cleavage embryos was found (p<0.0001) in a generalized additive model (GAM). Solid red line represents the smooth cure fit between variables. Blue bands represent the 95% of confidence interval from the fit. All adjusted for female age, BMI, weight, infertility factors, duration of infertility, AMH. bFSH. AFC, the proportion of POR and PCOS.

**Figure 4:**
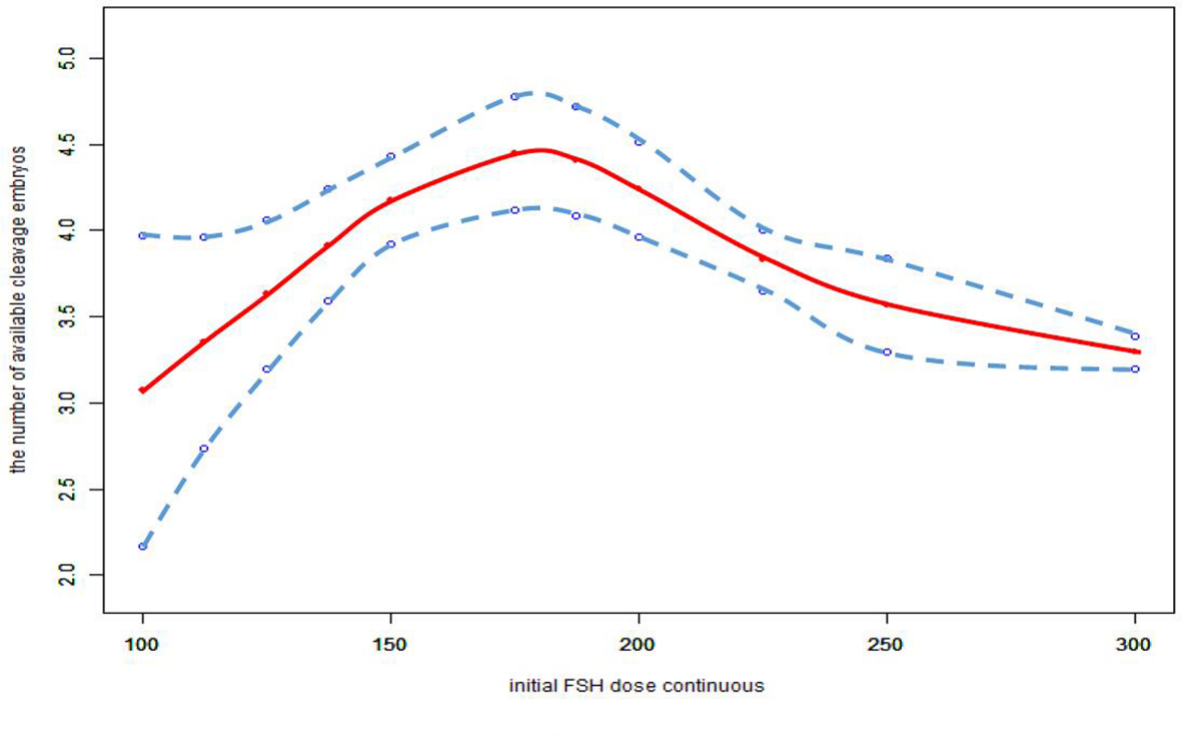
Association between the initial FSH dose and the number available cleavage embryos on day3 in the cycles in which FSH dose was not adjusted. A threshold, nonlinear association between the initial FSH dose and the number available cleavage embryos was found (p<0.0001) in a generalized additive model (GAM). Solid red line represents the smooth cure fit between variables. Blue bands represent the 95% of confidence interval from the fit. All adjusted for female age, BMI, weight, infertility factors, duration of infertility, AMH, bFSH, AFC, the proportion of POR and PCOS.

We also found that the relationship between total FSH dose and the number of available cleavage-stage embryos was nonlinear (**Figure 3**). The relationship between the initial FSH dose and the number of available cleavage-stage embryos was nonlinear in the FSH-unadjusted cycles (**Figure 4**).

## Discussion

The most important finding of this study was that the initial FSH dose was negatively correlated with the number of available cleavage-stage embryos, independent of female age, infertility factors, BMI, AMH, bFSH, AFC or the proportions of patients with POR or PCOS. A smoothed curve was fitted that showed an increasing and then decreasing relationship between the initial FSH dose and the number of available cleavage-stage embryos. The dose at which a saturation effect of the initial FSH dose was reached was 200 IU per day, after which the number of available cleavage-stage embryos decreased by 0.7 for every 75 IU increase.

We first analyzed the initial FSH dose as a continuous variable and identified the relationship between the initial FSH dose and the number of available cleavage-stage embryos. In previous studies, more than two doses of gonadotropins (including human menopausal gonadotropin) were investigated showing that the number of oocytes recovered increased with increasing doses, but the highest dose either did not increase or was detrimental to oocyte retrieval and clinical pregnancy rates or live birth rate (22–26), or no significant differences were found for fertilization rate, number of embryos formed and cryopreserved, and pregnancy rates between groups in women with a normal ovarian reserve (27). Increasing patient age and diminished ovarian reserve are associated with a poor response to COS. A meta-analysis on individual patient data (28) demonstrated that AFC and AMH clearly add to age in predicting a poor response. Therefore, these factors are often associated with the need for higher doses of FSH (29). However, other studies concluded that high doses of FSH did not increase the number of oocytes retrieved, number of embryos and improve assisted conception outcomes in patients with either a decreased AFC (30, 31), decreased serum AMH concentrations (32), or increased maternal age (33, 34). The literature has mainly reported the relationship between FSH dose and the number of oocytes retrieved, the number of embryos available for transfer, the implantation rate, the pregnancy rate or the live birth rate, and all reports have compared two or more initial doses in groups. Our study obtained similar conclusions that the initial FSH dose was negatively correlated with the number of available cleavage-stage embryos.

Several retrospective studies with large sample sizes (4, 5, 35) showed a negative correlation between gonadotropin dose and live birth rate, suggesting that high doses of gonadotropins (total doses >3000 U and mean daily doses > 300 IU) can impair oocyte maturation (4). Our data suggest that the total FSH dose is proportional to the initial FSH dose. In verifying the reliability of our results, we came to the same conclusion that the total FSH dosage was negatively correlated with the number of available cleavage-stage embryos (**Figure 3**), and the total FSH dosage to reach a saturation effect was 1150 IU. It has been shown that the number of available embryos in one cycle is directly proportional to the cumulative live birth rate (36). Therefore, it is more clinically relevant to explore the relationship between the initial FSH dose and the number of available embryos.

Multiple regression analyses by previous studies (4, 5, 35) adjusted only for age, while other factors related to ovarian sensitivity were not adjusted. We adjusted the baseline characteristics of participants such as female age, BMI, weight, infertility factors, duration of infertility, and adjusted for indicators related to ovarian reserve such as AMH, bFSH, AFC, and the proportions of patients with POR and PCOS. Previous studies have not distinguished between COS protocols, and have included both GnRH-agonist and GnRH-antagonist protocols. It is known that GnRH-agonist protocols are fundamentally different from GnRH-antagonist protocols. This is because regulation of the hypothalamic–pituitary axis by GnRH-agonists alters follicular synchronization and follicular sensitivity to FSH, resulting in differences in FSH dosage between the two protocols. Our study included only the GnRH-antagonist protocol, making the results more reliable.

There are now more animal-based studies supporting the idea that high FSH dosage can impair oocyte quality (37–39), and increase the numbers of degenerated embryos (10). In human studies, there has been increasing evidence in recent years that high doses of FSH can be detrimental to oocyte quality. One study suggested that gonadotropin stimulation might decrease the proliferation of granulosa cells, and impair ovulation, potentially inducing meiotic errors in human oocytes (40). It has also been suggested that exogenous FSH might increase the aneuploidy rate in human embryos (41). One retrospective analysis (42) indicated that high serum FSH levels during an IVF cycle led to the abnormal synthesis and secretion of zona pellucida (ZP) proteins from granulosa cells, and impaired embryo development. We believe that exogenous FSH doses above a certain level may have increased egg aneuploidy, decreased the expression of certain genes related to egg quality, affected the secretory synthesis of granulosa cell ZP proteins, or even other still unknown factors affected egg quality and thus embryo quality, leading to a decrease in the number of transferable embryos. Therefore, in our study, the initial FSH dose was negatively correlated with the number of cleavage-stage embryos available after the initial dose exceeded 200 IU per day even after adjusting for female age, BMI, weight, infertility factors, duration of infertility, AMH, bFSH level, AFC, and the proportions of patients with POR and PCOS. However, the bio-effective concentration of FSH entering the follicle is related to the FSH dose but also to the patient’s own factors (e.g., weight, BMI, and ovarian sensitivity to the hormone). Thus, clinicians should not assume that the optimal FSH initial dose is 200 IU for every patient, so a stratified analysis based on patient characteristics (age, weight, and ovarian reserve) is needed to find the optimal initial FSH dose for different populations and to provide more detailed reference for clinicians. The interaction analysis will also be used to determine which of these factors has the greatest impact on the initial dose of FSH, and to identify the factors that ART clinicians should prioritize when determining this.

There were some limitations to our study. The initial FSH dose range was 100–300 IU, with 50.95% of patients starting at 300 IU and 43.63% of the study cohort had a POR. This distribution might have caused bias in the results. Therefore, further analysis of the optimal initial FSH dose in different populations should be produced.

Furthermore, we only considered the initial FSH dose, and did not consider the effect of subsequent dose adjustments. Cycles with FSH dose adjustment were not excluded from this study; the data included 6604 cycles with an unadjusted FSH dose, 1625 with incremental FSH doses, and 543 with decreased FSH doses. In the FSH unadjusted cycles, the conclusions were unchanged (**Figure 4**).

Our study did not distinguish between rFSH and uFSH. One study found that more oocytes were obtained with the same dose of rFSH than with uFSH (43); in short, different initial FSH doses might be required using different sources of FSH. In addition, the decision and adjustment of the FSH initiation dose assumes the drug is given as a single dose. The adjustment of the dose of rFSH can be 12.5 or 25 IU, but the adjustment of the uFSH is only 75 IU per dose. This could influence the decision of the initial FSH dose.

In the GnRH antagonist protocol, the initial FSH dose showed a curvilinear relationship with the number of available cleavage-stage embryos. When the dose reached a certain level, the number of available cleavage-stage embryos decreased with increasing doses (*P* <.0001). Therefore, in COS, ART clinicians should try to avoid unnecessarily increasing the initial FSH dose. However, clinicians should not blindly assume that the optimal FSH initial dose is 200 IU for every patient, but still need to consider the patient’s age, BMI, weight, ovarian reserve function (e.g., AMH and bFSH levels, AFC) that may affect ovarian sensitivity to determine the FSH initial dose. Further analysis of the optimal initial FSH dose in different populations should be used to provide ART clinicians with a reference.

## Data availability statement

The raw data supporting the conclusions of this article will be made available by the authors, without undue reservation.

## Ethics statement

The studies involving human participants were reviewed and approved by the Institutional Review Board of Chongqing Health Center for Women and Children. Written informed consent for participation was not required for this study in accordance with the national legislation and the institutional requirements.

## Author contributions

The present work was designed by XL. Data extraction and analysis were performed by XL and LP. YH, FL, WH, SX, SH, JL, XZ and XL participated in the data collection. GH and HY participated in revisions to the article. All authors have read and approved the final manuscript.

## Acknowledgments

We gratefully acknowledge all the staff of Chongqing Reproductive and Genetics Institute for their support and cooperation. We thank James M Cummins, PhD, Liwen Bianji (Edanz) (www.liwenbianji.cn) for editing the language of a draft of this manuscript.

## Conflict of interest

The authors declare that the research was conducted in the absence of any commercial or financial relationships that could be construed as a potential conflict of interest.

## Publisher’s note

All claims expressed in this article are solely those of the authors and do not necessarily represent those of their affiliated organizations, or those of the publisher, the editors and the reviewers. Any product that may be evaluated in this article, or claim that may be made by its manufacturer, is not guaranteed or endorsed by the publisher.
